# Introducing ACTFAiREST^2^ to implement online assessments amid COVID–19: a case study from a low resource setting

**DOI:** 10.1186/s12912-022-01135-2

**Published:** 2022-12-17

**Authors:** Naghma Rizvi, Kiran Mubeen, Shanaz Cassum, Hussain Maqbool Ahmed Khuwaja, Zeenar Salim, Kiran Qasim Ali, Dilshad Noor Ali, Khairulnissa Ajani, Pammla Petrucka

**Affiliations:** 1 Aga Khan University School of Nursing and Midwifery, Karachi, Pakistan; 2 Syracuse University, School of Education, New York, USA; 3 Aga Khan University, Network of Quality, Teaching and Learning, Karachi, Pakistan; 4 University of Saskatchewan, College of Nursing, Regina, Saskatchewan Canada

**Keywords:** Online assessment, COVID-19 pandemic, Nursing education, Faculty readiness, Undergraduate nursing program, Pakistan

## Abstract

**Background:**

Amid COVID-19, soon after the closure of academic institutions, academia was compelled to implement teaching and assessments virtually. The situation was not the same for all countries. This transition was much more challenging in low-resource settings like Pakistan, where the students were geographically distant with minimal connectivity. A private university in Pakistan instituted a systematic approach for ensuring quality assurance and reliability before launching online assessments amid the COVID-19. The purpose of this study was to reflect on the phased transition to online/remote assessments to facilitate continuous student learning through distance modalities during the pandemic.

**Method:**

To assist faculty in re-designing their assessments, a workshop was conducted which was based on the modified Walker’s nine principles. The principles coded as “ACTFAiREST^2”^ were introduced to ensure that the faculty understands and adapts these principles in designing online assessments. The faculty modified and re-designed their course assessments, from face to face to online modality and submitted their proposals to the Curriculum Committee (CC). To guide the process of approving modified and re-designed assessments, a checklist was adapted. All the pre and -post workshop assessment proposals were analyzed using a content analysis approach to ensure the alignment of course learning outcomes with the assessments.

**Results:**

A total of 45 undergraduate courses’ assessment proposals were approved by the CC after deliberations ensuring their applicability in a virtual environment. From the analysis of the course outlines and assessment proposals submitted to the CC, faculty made four key changes to their assessment tasks in the light of ACT FAiREST^2^ principles (a) alternative to performance exams; (b) alternative to knowledge exams; (c) change in the mode of assessment administration; and (d) minimizing the overall assessment load.

**Conclusion:**

This transition provided an impetus for the faculty from a low resource setting to build momentum towards improved and innovative ways of online teaching and assessments for future nursing education to adapt to the new normal situation. This development will serve as a resource in similar contexts with planned and evidence-based approaches for enhancing faculty readiness and preparedness for online/remote assessments.

**Supplementary Information:**

The online version contains supplementary material available at 10.1186/s12912-022-01135-2.

## Introduction

In higher education, curriculum, teaching, and assessment are considered as the three pillars of the education system, regardless of delivery modality (i.e., online or face to face). Chen labels these components as the “three legs of the classroom stool” and reminds us that each leg must be equally strong for the “stool” to remain balanced and supportive [1]. The global Covid-19 pandemic has caused large-scale institutional and behavioral ‘shock waves’ in various areas of human activity, including education [2]. In the higher education setting, the closure of campus-based activities affected the stability of each of the three legs. Amid policies of compulsory physical distancing and various government-issued stay-at-home directives, the imperative was to teach and assess all the students online [3, 4]. This situation forced the change from traditional classroom-based to a technology-driven dominant curriculum, pedagogy, led by faculty in a relatively short period.

The imperative for new approaches to teaching and learning were increasingly apparent [5]. This sudden transition from face to face to distance learning approaches posed challenges for both faculty and students [6]. This situation gave popularity to digital and online learning platforms. Due to massive and unexpected closures, numerous countries and communities were forced to seek quick fixes through uptake of different digital media, infrastructure augmentation, as well as faculty training and development [6, 7]. This quick transition revealed gaps and shortcomings in how online learning has or has not been adopted in educational institutions [2]. In a recent study conducted in 225 health sciences educational institutes, Hussain and colleagues [8] reported that pre-COVID, 19.5% medical colleges, 17.3% dental colleges and 5% Nursing schools were using online learning platform in their routine academic year. Hence, amid COVID, the uptake of digital learning platforms was increased.

Historically, there have always been barriers to the widespread adoption of digital learning platforms [9]. However, given the need to adapt to the current situation and, in times of pressing change, educators responded by finding ways and methods to convert challenges into opportunities [10]. In this changing time, many educators faced limited internet access and insufficient preparation for using virtual pedagogical tools having roots in the dominant traditional approaches to teaching, creating a reluctance, if not resistance, to accept innovation [11]. The situation was much more challenging for health sciences universities that deal with skills-based learning in their curriculum. For students enrolled in health professional institutions; a certain level of competence is required to be assessed for promotion to the next level. Conventionally these competencies are learned and assessed in either skills lab, simulated environment or in a faculty-assisted clinical setting. Hence, if inadequately assessed, this has implications on their future clinical practices required at advanced level. This face-to-face contact was not possible amid-COVID-19. In contrast, the developed countries were equipped with virtual simulation soft wares that could assess their students’ competencies at different levels of fidelity [12]. Hativa and Goodyear suggest that the changing nature of both the student body and available technologies requires academics to change their teaching approaches to achieve improved learning outcomes [11]. These efforts to rebuild ‘the three-legged stool’ must be undertaken as the emergency remote learning environment has been criticized for failing to adhere to sound pedagogical principles and best practices [3].

There is empirical evidence that teachers teach and assess how they were taught and evaluated [13], taking inspiration from their lived experiences. Meloncon argues that the professional identity of instructors is tied to their past face-to-face teaching, where they had a high level of expertise. If educators are changing teaching approaches, they need to redefine themselves in light of the change in class-scape [14]. Therefore, there was a paradigm shift for educators as they strive to acclimatize and reframe their past practices in response to the emerging educational context and educational consumers’ expectations. This shift has implications for assessment, one of the legs of ‘classroom stool’, because teachers are now expected to plan for teaching online and conducting online assessments.

Globally, some higher education institutions closed for a specific time, while others made a rapid transition to online teaching using technology to meet the curricular requirements without compromising students’ learning. However, managing assessments at a distance remained a challenge for faculty and students in these latter situations due to limited access to internet, online platforms, readiness, and preparedness, availability of technology, equipment, technical support along with faculty and students acquiring COVID infection [15]. A needs assessment revealed that the teachers needed clarity on how to assess students. For example what to consider when selecting and implementing online assessments, and the types of assessments that can be conducted online, while considering approaches assuming that the students will take the responsibility of appearing for the exams independently without any proctoring [16]. Students must make extra efforts to complete their assignments [16] and demonstrate their trustworthiness in an online environment, which is also one of the vital features in professional nursing practice.

A private university in Pakistan instituted a systematic approach for ensuring quality assurance and reliability before launching online assessments amid the COVID-19. The faculty at the Aga Khan University School of Nursing and Midwifery in Pakistan (AKUSONAM-P) faced many of these challenges during the pandemic. This paper presents a case study that reflects the phased transition to online/remote assessments to facilitate continuous student learning through distance modalities during the COVID-19 pandemic. This phased transition included a needs assessment survey from faculty, adaptation of Walker’s (2007) nine principles to re-design their assessments (these principles were later coded with the acronym ACTFAiREST^2^), modifications in assessments proposed, approved and implemented based on ACTFAiREST^2^.

## Methods

**Fig. 1:**
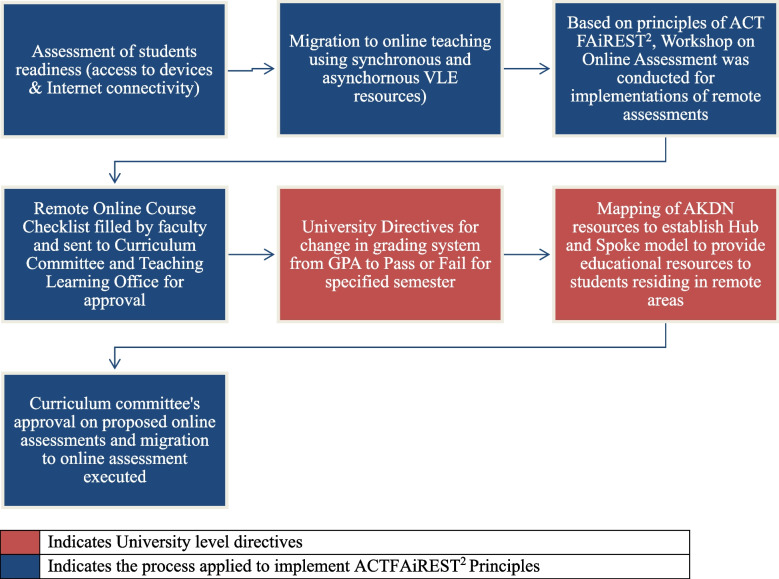
Stepwise approach for migration to online assessment amid COVID - 19

This case study investigated the processes at the undergraduate nursing programs at the AKUSONAM-P involved in the rapid transition of face-to-face educational assessments into online remote assessments amid the COVID-19 pandemic (Fig. 1). The semester had completed 10 out of 17 weeks of instructions when the pandemic struck. The government suspended all educational and business activities with concomitant lockdowns and social distancing for public safety. The Higher Education Commission for Pakistan [17] directed all public and private universities to stop on-campus instructions and rethink ways to teach students remotely. HEC released series of guidelines related to remote and online teaching learning and assessment during COVID-19. HEC directed its Technology Support Committee to assist universities with preparation of connectivity map to identify problem areas, collaborate with service providers, and arrange preferential access to educational materials and websites available at hec.gov.pk.

AKUSONAM-P committed to providing quality learning experience to its baccalaureate, masters and PhD nursing students (total 620 students), declared a mid-semester two-month break and stopped educational activities for undergraduate students because of their varied geographical distribution and high student volume. During this time, the entity undertook a student survey through phone calls and WhatsApp to determine accessibility of the students in terms of internet connections, which revealed that around 45% of students reside in the remote areas of northern Pakistan and had no to poor internet access or devices to partake in digitally based course instruction. In contrast, the remaining 55% faced relatively fewer connectivity issues. This situation resulted in two cohorts, one in continuous communication with faculty via email and WhatsApp communications, and the other cohort receiving communication through the university’s representatives at the designated hubs, particularly in northern areas of Pakistan.

The University Registrar and Provost Office proactively initiated implementation planning as per HEC directives and directed all faculty to think of online teaching and assessment approaches in the emerging COVID crisis. Individuals were invited to submit their proposals on a specified template called Online Remote Readiness Checklist (ORRC) [18, 19]. The Teaching-Learning Undergraduate (TLUG) office of AKUSONAM-P requested all course coordinators to complete the ORRC and assigned the CC to review the proposed ORCC for the 45 courses and approve them based on the learning outcomes alignment with teaching-learning pedagogies and assessments. The University also issued a policy on change in semester grading during the pandemic to be fair and humane so that students can be graded pass or fail with a 60% cut off versus assigning a Grade Point Average (GPA).

Next, assessment of faculty readiness was gathered using a questionnaire (Table 1).

**Table 1:** Questionnaire for faculty readiness

1. To what extent do you feel prepared regarding switching to Online Assessment amid COVID pandemic? (Response on Likert scale of 1–5. 1` least prepared & 5 most prepared) 2. How well equipped do you feel to handle/troubleshoot/overcome issues related to technology? (Response on Likert scale of 1–5. 1` least prepared & 5 most prepared) 3. What is your apprehension level regarding switching to online assessments amid COVID pandemic? (Response on Likert scale of 1–5. 1` least prepared & 5 most prepared) 4. What are your overall reflections regarding online assessments in this new normal situation amid COVID 19? 5. What are some of your apprehensions/fears/concerns regarding online assessments amid COVID 19? 6. What issues/challenges/opportunities do you anticipate during planning and implementation of an online assessment that you are proposing in your course?	

to explore their perceptions, fears & opinions on switching to online course instruction amid COVID. The questionnaire was responded by 65% of the faculty teaching during that semester. Over half (52%) of the faculty members felt prepared to online instruction, while the remainder felt less prepared. Moreover, 40% of the faculty members felt apprehensive about designing and implementing assessments, although they were willing to switch to the online assessment mode.

A virtual workshop was offered for all AKUSONAM faculty members on “Online /Remote Assessment “ to build faculty capacity and confidence in redesigning their course assessments for online delivery. In particular, the workshop aimed to support faculty members in:

- Identifying principles of assessment for students of SONAM-P in the time of rapid transition to online/remote teaching
- Deciding appropriate assessment methods according to the learning outcomes of the current courses and students’ connectivity and device access
- Using appropriate digital tools for the chosen assessment methods
- Identifying the possible challenges of online and remote assessment and how these might be mitigated

This three-hour workshop was facilitated by a team of AKUSONAM experts involving the entity’s Assistant Dean, Teaching Learning, the CC chairs, and educational experts from the Quality Teaching Learning Network (QTL_Net) at AKU. Faculty members were briefed on different online assessment strategies and Walker’s (2007) nine principles to re-design their assessments [20]. The adapted principles were coded with the acronym ACTFAiREST^2^ in Table 2 to help them remember to design online assessments that are fair, aligned with course outcomes, and valid and reliable. In the workshop, faculty members worked in small groups. They prepared guidelines for various online assessments, such as oral presentations, VODCAST, short essay questions, reflective paper, discussion forum, and lab-based skill assessment, both for high bandwidth and low bandwidth internet access students, in synchronous or asynchronous modes.

**Table 2:** Brief description of ACTFAiREST^2^

Principle	Strategies to consider for Implementation
1. **A**lign with curriculum & learning outcomes	Matching of an assessment to each learning outcome.
2. **C**larity of instructions & wording	Clearly stating detailed guidelines. Standardize the assessments. Well-developed rubrics for assessment. Use variety of ways (such as video, audio, written) to provide guidelines
3. **T**imeliness of meaningful feedback	Provide constructive feedback to students in timely manner
4. **F**air & transparent marking strategies	Share grading policies with students at the beginning of the course
5. **A**ccessible & accommodating assessments a. **i**nnovate and imagine	Make assessment intuitive and engaging Offer choices in how students meet learning outcomes
6. **R**ange of Assessment Tasks	Provide range and variety of assessment tasks
7. **E**valuate Objectively	Develop competency-based assessment
8. **S**tudent Levelling	Not all students are at same level; do not assume homogeneity
9. **T**iming & Time	A balance and integration between formative and summative assessments.
10. **T**est on Point not for Tech	Assessments should be graded on the content not the technical competency. Give clear instructions or demonstration and use practice time for any technical competence required in completing an assessment.

After the workshop, faculty members were asked to revisit their ORRCs by applying the ACTFAiREST^2^ principles. Later, the CC assessed the modifications suggested by course coordinators for each course. Finally, using HEC guidelines and considering the contextual factors of barriers to online education in Pakistan, the CC analyzed the modifications in assessments proposed by faculty members and assessed the principles of ACTFAiREST^2^ guidelines applied in each course for approval and execution.

A checklist adapted from the University of Calgary was used [21]. This checklist was a benchmark to ensure appropriateness of the proposed online assessments in terms of its implementation and feasibility (Refer Additional 1). Conventionally, the CC used to approve proposed and revised face-to-face assessments strategies, whereas the checklist facilitated approval of proposed online assessment amid COVID ensuring the principles of fairness and equity without compromising on standard practices of teaching learning and assessment.

### Data analysis

A content analysis approach was applied to determine the approaches used by faculty members to modify pre-COVID face-to-face assessments to online/remote assessments. Faculty learnt the ACT FAiREST^2^ principles and incorporated them into their respective online assessments to support distressed and distanced students’ learning amid the pandemic. Post-workshop, faculty were instructed by the CC to develop and present two different assessment options, one in synchronous mode for those students who had internet facilities, and could access learning management system, and other in an asynchronous mode for those who did not. This was done to assure that ‘all students’ with access to high, low, or no bandwidth internet services, could undertake the assessments tasks and complete their course requirements. Documents submitted to the CC; the course outlines, assessment proposals developed in the light of ACT FAiREST2 principles, and the ORRC checklist, were reviewed using a systematic approach of content analysis [22]. Each document was read and re-read to identify similar modifications across different courses. Each modification in a course was given a code. The similar codes were identified based on the type of alternative assessment strategy proposed for a knowledgebase or skill-based exam and the modifications made form face to face to online modality. This was considered keeping in mind the type of assessments and its alignment with the course learning outcomes. Later all the similar codes were condensed to form categories to reflect the types of modifications made and approved. The categories were translated into four broad themes. These themes reflect the types of changes made in the assessment tasks during the phased transition.

## Results

The themes are as follows: (a) Alternative to performance exams; (b) Alternative to knowledge exams; (c) Change in the mode of assessment administration; and (d) Minimizing the overall assessment load. These changes are described below:

- Alternative to performance/skills-based exams
  During the workshop, faculty who taught predominantly skills-based components or bedside nursing reported difficulty conceptualizing alternative assessments that gauge students’ skills and attitudes towards patients in this new environment. However, these alternative assessments were not considered as ‘pure’ reflections of learning outcomes; hence, they were not direct replacements of the existing practical assessments. Nevertheless, attending to the current pandemic situation and social distancing protocols, the existing approaches were re-imagined yielding new and innovative assessment tasks. To complete the clinical objectives, faculty members used virtual reality simulation softwares such as Cyber Patient and Body Interact however, for assessing the skills/practical aspects; other modalities were used. For example, in the Health Assessment course for the 2nd year BScN students, a performance assessment aiming at assessing the ability to perform interviewing and physical assessment of adult client and utilizing techniques of observational and physical examination, was replaced with a VODCAST. The students with stable internet and advanced camera devices were then asked to record a 5- minute video recording conducting a physical examination on any of their family members and upload a 5-minute video as an assessment product. Alternatively, students with basic camera and internet devices were given the option to capture 20 photographs and upload these as a presentation or submit it through a pen drive. In another example, in the critical care nursing course, the double jump exam, where the clinical performance and reasoning skills are assessed in real hospital settings, was replaced with a case-based scenario accompanied by the written questions. The weightage of the assessment item was increased from 30 to 45% and students were given 48 hours to complete the assessment. A third example occurred in the biochemistry course. In that course the lab-based performance evaluation of biochemistry was replaced with the videos of lab performances which was shared with the students. Moreover, it was followed by a post quiz, where students had to answer scenario-based questions and submit them through the learning management system or via a memory stick. Alternative assessments were designed keeping in mind that the students are not being graded for their technology skills or availability; hence, multiple alternatives were provided for high- and low-bandwidth students. One could argue that there were trade-offs in making the assessments accessible and accommodating for students while reflecting the core learning outcomes.
- Alternative to knowledge exams
  Before the pandemic, knowledge-based exams were administered through paper-pencil tests and were proctored through an in-person testing environment to ensure integrity and fairness in the assessment. These in-person proctored exams were replaced with open book and take-home exams, some of which were time-bound. However, there was greater flexibility in terms of submission of examination (i.e., students were given the opportunity to complete the exam within an hour slot in 24–48 hours from when the exam has been posted). This flexibility accommodated the limited or inconsistent access that the students experienced with internet facilities in certain regions and at certain times across Pakistan. Some courses changed their questions from the ‘search-able’ answers to those that are higher-order questions to restrict the opportunities that student’s might ‘google’ the answers at the time of attempting the test. For example, in pre-COVID, the Community Health Nursing II course conducted a time-bound multiple-choice (MCQs) final exam. Amid COVID, several new case studies were formulated in which students were expected to integrate several concepts from the course to respond to Short Answer Questions (SAQs). Similarly, in the Nursing Research course, the individual final exam was replaced with a group assignment developing a proposal. Students were expected to integrate the concepts they had learnt throughout the course. The groups were assigned different scenarios developed by the course team. These scenarios were based on different aspects of promoting nursing research during the pandemic. Resultantly, these amendments ensured assessments that required students’ higher-order thinking. An honor code was included with the test to explain what would be defined as cheating and plagiarism.
- Change in mode of assessment administration
  There was a shift in how exams were administered. Before COVID, the most popular method was paper and pencil exams administered in an examination hall setting, without allowing students access to the internet and their technological devices. Amid COVID, however, the examination mode changed to online exams. For example, in a biostatistics course, pre-COVID testing included a time-bound open book class-based assignment, which was replaced with home-based open book assignments with a one-week submission deadline. Similarly, in the Teaching-Learning Principles & Practices course, typically, students delivered a teaching presentation in the group to actual clients in the community. Still, amid-COVID, they were asked to develop a vodcast of their teaching presentation. Similarly, pre-COVID Public Speaking/Oral Presentation assignments were converted to VODCAST assignments in the English Language course. To mitigate the issue of low bandwidth, the students were expected to deliver teaching on any one objective of their teaching plan and limit their video to 5 min to facilitate data transfer. Since, students’ clinical experiences were paused for some time, typical assignments based on the identified patient from the clinical area (hospital) and scholarly/analytical papers could not be conducted. Instead, the live cases were replaced with the simulated patients and students were expected to write paper-based assignments aligned with the standard CC approved rubrics. Thus, there were real challenges in the assessments following the principles of ‘fairness’ and ‘equity’ and decisions were made based on what instructors considered best, given the circumstances.
- Minimizing the overall assessment load
  There were 45 courses that were reviewed and approved during this process. Some examples of the modifications done in the assessments are shared in Table 3. In certain courses, the percentages of assessments allocated pre-COVID were revised to reduce the number of assessments per course. The rationale behind this exercise was to lessen the overall number of assessments as students were left with less time to complete the semester and to ensure that students received due credit for their efforts for the modified assignment as it was remotely attempted. For example, the Critical Care Nursing course in Year IV BScN had the double jump exam, which carried 30% of the weightage of the total course, replaced by an open book clinical scenario followed by SAQs with a weightage of 45%. Because students had less time to complete their final assessments, the previously conducted assessment weightages were increased to reduce the load of final exam or assessment. Final exams were waived off in courses with fewer credit hours that had completed 70% of the assessments. This reduced the overall students’ assessment load at semester end. Throughout the reallocation of weightages, it was ensured that the assessments were aligned with the course learning outcomes. Hence, if the pre-COVID assessment of the course completely fulfilled the learning outcomes of that course, the remaining assessments were removed, and the weightages of already conducted assessments were re-allocated. For example, the final exam of the two courses (both having 2 credits) were pending during COVID. Both the courses, Culture, Health and Society and Nursing Theories, had project-based assessments that aimed to assess the application of the theoretical concepts. As the applications of the theoretical concepts were already assessed pre-COVID, final exams were not taken, and the assignment percentages were re-allocated to the assessments already conducted.

**Table 3:** Examples of modified course assessments amid COVID-19

Name of course (No. of Credits)	Pre-COVID assessments	Approved Amid COVID assessments
Teaching Learning Principles and Practices **(3.0)**	Teaching Project **40%** • Lesson Plan 5% • Teaching Presentation 15% • Teaching Report 20% Reflective Log **45%** Course participation **15%**	Teaching Project **40%** • Need Assessment & Lesson Plan 20% • Teaching Presentation (VODCAST) 20% Reflective Log **45%** Course Participation **15%**
Community Health Nursing I (6 credits - 2.5 theory, 2.5 clinical*, 1 skills)	Critical Incident Analysis 25**%** Clinical Portfolio **10%** Midterm Exam **30%** Final Exam **35%**	Critical Incident Analysis 25**%** Clinical Portfolio **10%** Midterm Exam **30%** Final Exam (Open Book case study) **35%**
Biochemistry for Nurses (3.0 credits - 2.0 theory, 1.0 lab)	CAT 1 **15%** CAT II **15%** Lab Project **20%** Lab Performance Evaluation **20%** Final Exam **30%**	CAT 1 **15%** CAT II **15%** Lab Project **20%** Video-based Quiz **20%** Final Exam (Open book SAQs) **30%**
Health Assessment I (2.0 credits - 1.0 theory, 1.0 skills)	Course Participation **10%** Online Midterm exam **25%** Final Exam **25%** Performance Exam **40%**	Course Participation **20%** Online Midterm exam **30%** Open Book Final exam **30%** Performance Exam (VODCAST) **20%**
Culture, Health & Society (2.0)	Cultural Assessment Questionnaire (Group) **30%** Reflection (Individual) **30%** Final Exam **40%**	Cultural assessment questionnaire (Group) **50%** Reflection (Individual) **50%**
Critical Care Nursing (Theory: 2.5, Clinical: 4.0, Skills/Lab: 0.5)	Midterm Exam **30%** Final Exam **40%** Double Jump Exam **30%**	Midterm Exam **30%** Online Final Exam **25%** Open Book Case Study **45%**
Nursing Research (3.0)	Midterm Examination **25%** Quantitative Critique (Group Presentation) **20%** Qualitative Critique (Group Presentation) **20%** Final Examination **35%**	Midterm Examination **25%** Quantitative Critique (Group Presentation) **20%** Proposal Development Scenario-Based (Group) **55%**
Introduction to Nursing Theories (2.0)	Quiz 1 **20%** Quiz 2 **20%** Group Presentation **30%** Quiz 3 **30%**	Quiz 1 **30%** Quiz 2 **30%** Group Presentation **40%**

## Discussion

The implementation phase of online assessment in our context was highly challenging; however, the team constantly considered AKU’s mission and core values framework, Impact, Quality, Relevance and Access (IQRA, which in Arabic means “Read”) as the guiding principle for decision making [23]. The workshop and post-workshop support processes, such as feedback on individual course outlines and presentation of the course outlines in the CC, were important for faculty to reconsider the assessments in the light of ACTFAiREST^2^ principles.

Faculty made attempts to innovate and re-imagine the existing assessment tasks, put objective measures, time it according to the remaining course duration and crises situation, match assessment to student cognitive level and content, develop clear and concise instructions and, above all, generate the assessments options for both have and have not audiences. It is important to realize that presentation of ACTFAiREST^2^ principles and having several discussions did not provide faculty a ‘silver bullet’ solution to the challenges they faced during the design and administration of the assessments. Analysis of various courses made it apparent that faculty used these principles to adapt their assessment design decisions; however, rapid application of these principles in re-designing assessments fell short of the ideal which would have allowed a slow and planned approach.

The major challenge in applying ACTFAiREST^2^ in our context was the short timeline in implementing the entire process. Establishing feasibility for hub and spoke models by the university consumed most of the time. Therefore, when the students received the data package, very little time was left to complete the semester. Due to the shorter timeline, they may have not received feedback on formative assessment that could have helped them in improving on their summative assessments. The CC proactively and mindfully dealt with the situation by minimizing the assessment load without compromising the course learning outcomes. Conclusively, attempts were made to act ‘fair’ with the students and help reduce cognitive load during the times of collective trauma, without compromising the quality of learning. Recent evidence from Saudi Arabia confirms that the alternative methods of assessments, such as written assignments, are preferred by faculty members and students, provided that the students receive detailed guidelines on what is expected [24].

Another challenge in implementing online assessment in undergraduate nursing curricula, related to the concentration of more than half of scholastic credit hours to the clinical components, was assessing the skills and practical components across courses. Faculty teaching clinical and skills-based courses found it challenging to design e-assessments that reflected the outcomes and judge students’ skills while doing performance based or clinical exams. This situation gave faculty members the opportunity to think out of the box and explore several tools of virtual reality simulations, such as Cyber Patient and Body Interact, in which students’ performance can be assessed through more reliable measures. A similar approach was taken by the Texas Woman’s University, where the undergraduate and graduate nursing students were offered virtual simulations to replace clinical rotations. Overall, the students expressed positive learning experiences in virtual simulations [25]. Shehata and colleagues presents practical tips for virtual meeting software to virtually plan, execute, and assess clinical outcomes [26].

According to the ACTFAiREST^2^ principles, choices can be given to students for choosing an assessment modality. Given our context, the faculty identified some limitations in the implementation of this principle. They shared concerns over the unequal accessibility of high bandwidth internet access among students; for instance, students with better internet access would gain better learning experiences than the cohort with limited internet access. Therefore, the committee mutually agreed that the same assessments would be administered/delivered for both cohorts to be fair.

The connectivity constraints mentioned above posed limitations for conducting online synchronous assessment with proctoring and invigilation. Although proctoring could have been introduced to students who had access to the internet and devices, to be fair with all, proctoring was not used across the board. Because the students were scattered across the country, invigilation in all hubs could not be ensured. A recent pilot study conducted on the feasibility of proctoring online assessments stressed the need for extensive technology and human resources to ensure smooth implementation of proctored exams in limited resource contexts like Pakistan [27]. Therefore, Open Book Exams (OBEs) appeared the best option under these circumstances. Zagury-Orly and Durning affirmed that due to the limited possibility of Case Base Exams during a pandemic, now is the time for us to strategically explore the use of online Open Book Exams in health sciences education [28]. Questions that stimulate critical thinking and logical reasoning were developed to avoid recall-based response and to ensure integrity. Academic honesty was further strengthened by expecting students to submit their work through the Learning Management System (LMS) and with references. Plagiarism checks were built-in in the LMS that automatically monitors possible plagiarism when submitting assignments.

Our faculty were highly concerned for exposing their question bank by sending validated and reliable banked questions when assessing students remotely. Therefore, for this period, time bound password protected open book assessments were sent to students through offline data packages.

Besides the above challenges, one of the unprecedented obstacles in operationalizing remote assessment was the weather conditions of the country. The southern region, where the university is situated, experienced heavy rainfall followed by urban flooding which badly affected students’ and faculty members’ internet connectivity due to major power shutdowns. Similarly, the northern region was also affected by heavy rains and landslides, which affected students’ commutation to their hubs to submit their assignments timely through LMS. Despite meeting close timelines to complete the semester objectives, the Teaching Learning Office was flexible. It extended the assignment submission timeline for students isolated due to either very unstable or no connectivity. A recent study conducted in Southern California affirmed that the faculty believed in adjusting expectations about assignments like changing letter grades to PASS/FAIL and extending deadlines helped students cope with academic stress [29]. In two other undergraduate programs with a lesser number of students (i.e., Post RN BScN and BScM), 100% of students had access to the internet and devices; hence, the online synchronous modality was the preferred method of assessment. During synchronous exams, students were asked to keep possible backups ready in case of shutdown of internet connectivity.

### Impacts and implications

#### Faculty

##### Awareness

As the Covid-19 events unfolded, faculty became more willing to explore alternatives in keeping the program functioning. These options often gave more control to the students for selection of assignment type/assessment type, which had not been the norm pre-Covid-19. As these approaches evolved, many faculty gained a deeper understanding of the conditions of the students and from where they have come, which may change future interactions with ‘out of Karachi’ students on first arrival into our program. This experience has reminded faculty at a much deeper level, about the constraints and the barriers faced by these newcomer students.

##### Openness to emerging teaching practices

As the situation evolved, the dialogues shifted to possibilities rather than barriers in delivery of our program. Many of those possibilities, especially in the clinical courses, are being considered as part of the next curriculum design activities. The fears respecting not meeting program outcomes are slowly being displaced with tentative steps towards a more open, learner-centred curriculum.

##### Technology Embeddedness

In conjunction with the ‘Openness to emerging teaching practices’, technology as integral rather than subsidiary to our efforts was made apparent during this period. Many faculty recognized that these Covid-19 teaching approaches were appropriate in a twenty-first century nursing program for twenty-first century learners who tend to have extensive skills (and preferences) in technology, social media, and collaborative contributions. For faculty the pressure for technological embeddedness created opportunities for shared experiences and co-learning of tools, strategies, and assessment techniques. The experience validated and even catalyzed efforts to look at technology as a faculty extender. For example, during this Covid-19 response efforts to use the simulation software seems to have potentiated previous efforts in simulation and virtual reality which were under way.

#### Students

##### Gaining voice

As Covid-19 emerged and persisted students became more vocal on their challenges and needs to ensure success. Students spoke of local and generic needs, such as bandwidth, transportation, and resources, in order to ensure that their learning and assessment opportunities were equitable and achievable. It is unclear how and if this advocacy will continue in the next phase of our program, but they were engaged in moving the program forwarding throughout these unusual times.

### Strengths and limitations

Although these findings are from a large, private and highly resourced teaching university in terms of IT support, strong administration, qualified faculty and, they cannot be generalized to other universities that are comparatively less resourced. The entire initiative utilized programmatic funds and no external grants were obtained or available. Hence, the university was depending on its own resources that made each one of us resilient to adapt this change.

#### Future research

A scoping review on COVID – 19 related initiatives can be conducted using the ACTFAiREST^2^ Framework to see how these innovations respond to the global challenge of nursing education. In addition, a two-year follow-up study will determine what innovations are retained or embedded in the curriculum and/or need modification.

## Conclusion

During the journey, faculty were considerate and vigilant in making the decisions around assessment during the COVID period. However, further deliberations, discussions, and reflections are needed to continue with the ideas presented in the workshop. Nevertheless, having a set of principles and discussions did provide a platform and due support needed for faculty to rethink their assessments during times of collective trauma. Notwithstanding the foregoing, the walk towards integrating alternative assessments as part of the learning process, reflecting the principles, and being authentic to reflect the real-life practical situations that graduates will face in their practice, during or post-pandemic, is a road less travelled in nursing education. However, it is an opportunity and a catalyst to change for optimizing our future in nursing education if we travel it together and build momentum towards improvement and innovation.

## Supplementary Information

**Additional file 1.** 

## Data Availability

The datasets generated and analyzed during the current study are not publicly available because the data included documents such as course grids, which, as per institution’s policy are university’s intellectual property and are considered confidential, therefore, should not be shared outside university. However, it can be made available from the corresponding author on reasonable request.

## Abbreviations

AKUSONAM-P: Aga Khan University School of Nursing and Midwifery in Pakistan
TLUG: Teaching-Learning Undergraduate
GPA: Grade Point Average
CC: Curriculum Committee
SONAM: School of Nursing & Midwifery
ORRC: Online Remote Readiness Checklist
HEC: Higher Education Commission
QTL_Net: Quality Teaching Learning Network
AKU: Aga Khan University
MCQs: Multiple Choice Questions
SAQs: Short Answer Questions
BScN: Bachelor of Science in Nursing
BScM: Bachelor of Science in Midwifery
IQRA: Impact, Quality, Relevance, Access
OBE: Open Book Exam
LMS: Learning Management System
