# Supplementary material for: Introducing ACTFAiREST2 to implement online assessments amid COVID–19: a case study from a low resource setting
Source: BMC Nurs. 2022 Dec 17;21:361. doi: 10.1186/s12912-022-01135-2 (PMC9758660; doi:10.1186/s12912-022-01135-2)
Supplement: Supplementary file 1 — Additional file 1. [file 12912_2022_1135_MOESM1_ESM.docx]

**Appendix 1**

**Online Assessment & Alignment Checklist**

Use the following checklist to review assessment and alignment plan.

| **Criteria** | **Findings** | **Comments (if any)** |
| --- | --- | --- |
| Is each assessment clearly aligned with at least one course learning outcome? | 1. Yes 2. No |  |
| Do the assessments emphasize (check all that apply) | 1. foundational understanding 2. critical thinking/reflection 3. applying theories and concepts 4. innovation |  |
| Do the assessment weightings reflect the degree of work required and the importance of the work? | 1. Yes 2. No |  |
| Can the assignments be reasonably completed within the given time frame? | 1. Yes 2. No |  |
| Is there a variety of assessment strategies to provide students opportunities to demonstrate learning in different ways?  *Note:* This will depend on nature of course | 1. Yes 2. No |  |
| Are there subsequent assessment opportunities for students to apply feedback? | 1. Yes 2. No |  |
| Are the number and type of assessments manageable in terms of grading and feedback workload for the instructor (consider the number of students and Faculty)? | 1. Yes 2. No |  |

Adapted from: University of Calgary
